# Self-efficacy of advanced cancer patients for participation in treatment-related decision-making in six European countries: the ACTION study

**DOI:** 10.1007/s00520-023-07974-2

**Published:** 2023-08-08

**Authors:** Berivan Yildiz, Ida J. Korfage, Luc Deliens, Nancy J. Preston, Guido Miccinesi, Hana Kodba-Ceh, Kristian Pollock, Anna Thit Johnsen, Johannes J. M. van Delden, Judith A. C. Rietjens, Agnes van der Heide

**Affiliations:** 1 Erasmus MC, University Medical Center, Rotterdam, The Netherlands; 2 End-of-Life Care Research Group, Vrije Universiteit Brussel (VUB) & Ghent University, Brussels, Belgium; 3 International Observatory on End of Life Care, Division of Health Research, Lancaster University, Lancaster, UK; 4 Clinical Epidemiology, Oncological Network, Prevention and Research Institute (ISPRO), Florence, Italy; 5 University Clinic of Respiratory and Allergic Diseases Golnik, Golnik, Slovenia; 6 School of Health Sciences, University of Nottingham, Nottingham, UK; 7 Department of Psychology, University of Southern Denmark, Campusvej, 55 Odense, Denmark; 8 Department of Oncology and Palliative Care, Zealand University Hospital, Roskilde, Denmark; 9 Julius Center for Health Sciences and Primary Care, University Medical Center Utrecht, Utrecht, The Netherlands

**Keywords:** Self-efficacy, Decision-making, Advanced cancer, Coping, Support

## Abstract

**Purpose:**

Many patients prefer an active role in making decisions about their care and treatment, but participating in such decision-making is challenging. The aim of this study was to explore whether patient-reported outcomes (quality of life and patient satisfaction), patients’ coping strategies, and sociodemographic and clinical characteristics were associated with self-efficacy for participation in decision-making among patients with advanced cancer.

**Methods:**

We used baseline data from the ACTION trial of patients with advanced colorectal or lung cancer from six European countries, including scores on the decision-making participation self-efficacy (DEPS) scale, EORTC QLQ-C15-PAL questionnaire, and the EORTC IN-PATSAT32 questionnaire. Multivariable linear regression analyses were used to examine associations with self-efficacy scores.

**Results:**

The sample included 660 patients with a mean age of 66 years (SD 10). Patients had a mean score of 73 (SD 24) for self-efficacy. Problem-focused coping (B 1.41 (95% CI 0.77 to 2.06)), better quality of life (B 2.34 (95% CI 0.89 to 3.80)), and more patient satisfaction (B 7.59 (95% CI 5.61 to 9.56)) were associated with a higher level of self-efficacy. Patients in the Netherlands had a higher level of self-efficacy than patients in Belgium ((B 7.85 (95% CI 2.28 to 13.42)), whereas Italian patients had a lower level ((B −7.50 (95% CI −13.04 to −1.96)) than those in Belgium.

**Conclusion:**

Coping style, quality of life, and patient satisfaction with care were associated with self-efficacy for participation in decision-making among patients with advanced cancer. These factors are important to consider for healthcare professionals when supporting patients in decision-making processes.

**Supplementary Information:**

The online version contains supplementary material available at 10.1007/s00520-023-07974-2.

## Introduction

Patients with advanced cancer often face complex treatment decisions that can have important consequences in terms of quality and length of life. Shared decision-making has been acknowledged as the preferred model to engage and support patients in the decision-making process about diagnosis, treatment, and care [1, 2]. Initiatives targeting shared decision-making are growing and are advocated internationally by key figures such as patient representatives, policymakers, and hospitals [1]. Shared decision-making requires active participation from both health care provider and patient as they have to collaborate in considering relevant treatment options, taking into account the goals and values of the patient. An important aspect for physicians in the decision-making process is to communicate and behave in a way that facilitates the patient to express him or herself, speak openly, and ask questions about their care and treatment options [3, 4].

It has been suggested that patients who participate more actively in decision-making processes have better physical and mental health and tend to be more satisfied with their care than patients with a less active role [5, 6]. In addition, patient participation in decision-making improves the patient-physician relationship and communication [7, 8]. However, although many patients prefer an active role [9, 10], it is challenging for patients to adequately participate in decision-making about treatment and care [11]. Several studies have shown a difference between patients’ preferred and actual roles during decision-making [11, 12]. For patients, an important aspect for participation in decision-making according to their preferred role is self-efficacy in communicating with physicians [4]. Self-efficacy refers to individual’s confidence or belief to carry out a specific task in order to reach a desired outcome [13]. In the interaction with physicians, self-efficacy is the self-perceived ability of patients to gather medical information from physicians and to ensure that their health-related concerns are identified and addressed [14]. Self-efficacy regarding decision-making is associated with greater participation in treatment-related decision-making [15], such as asking questions, and better disease-specific health-related quality of life [16].

Self-efficacy in decision-making can affect and be affected by elements of the patient-physician interaction, including information exchange, trust, satisfaction, and understanding instructions and treatment options [17, 18]. The more the patient has experienced being listened to and having questions answered, the more likely the patient is to have higher levels of self-efficacy [19]. Furthermore, in a study among patients with prostate cancer, those who were less satisfied with their care, or had more symptom distress were more likely to have a low level of self-efficacy in interacting with physicians [20]. In non-oncology settings, it was suggested that patients provided with good verbal support by their physician (e.g., information about symptoms and strategies to manage these) may have higher levels of self-efficacy in decision-making [21, 22]. Patient-related factors such as health literacy, socioeconomic status, age, religious beliefs, and coping with cancer have also been linked to self-efficacy [14, 23–28]. In addition, clinical characteristics such as poor clinical performance status have been associated with less confidence in making decisions among patients with lung cancer [29]. All these studies have, however, been conducted among specific patient populations, such as older people, or within particular types of cancer, including breast cancer or prostate cancer, and are, thus, limited to either male or female subjects. Less is known about self-efficacy regarding decision-making among patients diagnosed with highly prevalent cancers that affect both males and females, such as lung and colorectal cancer.

A better understanding of factors associated with self-efficacy is important for supporting patients’ participation in the decision-making process. The aim of this study was to explore whether sociodemographic and clinical characteristics, patient-reported outcomes (i.e., quality of life (QoL) and patient satisfaction), and coping strategies were associated with self-efficacy regarding treatment-related decision-making among patients with advanced lung or colorectal cancer in six European countries.

## Methods

### Study design and setting

Baseline data were used from the ACTION study, a multicenter cluster-randomized trial to investigate the effects of an advance care planning (ACP) intervention compared to care as usual [30]. ACP is a formal process of communication between patients, relatives, and healthcare professionals about future care and promotes discussion of preferences [31]. Patients were recruited in pulmonology and oncology departments in academic and nonacademic hospitals in Belgium, Denmark, Italy, the Netherlands, Slovenia, and the UK. Written informed consent was obtained. The trial was approved by research ethics committees in all participating countries.

Recruitment took place between May 2015 and February 2018. Hospitals were randomized to the intervention arm, providing usual care and ACP, or the control arm, providing usual care. All patients with advanced lung (stage III or IV) or colorectal cancer (stage IV), WHO performance status 0–3, an estimated life expectancy of at least 3 months, and competence to give consent were eligible. When a care team considered patients eligible, they were asked to consider participation in ACTION. Patients who wanted to consider participation were contacted by the researcher team and provided with more information about the study. Patients in control hospitals were informed that the study focused on preparing patients for decision-making about care, and that they would receive usual care.

Due to the cluster-randomized study design in which patients knew beforehand whether they were recruited for the intervention group or the control group, and as the response rate was substantially lower in the intervention than in the control arm [32], patients in the intervention and control groups may not be entirely comparable. Since the response rate was substantially higher in the control group and, thus, data from this group was more reliable, we chose to only include baseline patient data from the control arm (care as usual) in the present study. Care as usual mainly consisted of receiving systemic treatment and related support as provided by the hospitals.

## Measures

Patients were asked to complete a written questionnaire at baseline, at 2.5 months, and 4.5 months after inclusion. The primary outcomes of the ACTION study were quality of life, assessed with 10 items of the European Organisation for Research and Treatment of Cancer (EORTC) emotional functioning item bank [33, 34] and symptoms (EORTC QLQ-C15-PAL [35]). Several secondary outcomes were coping (COPE, Brief COPE [36, 37]), satisfaction with care (items of the EORTC IN-PATSAT [38]), satisfaction with the intervention (9 study-constructed items), and shared decision-making (decision-making self-efficacy scale [39]). Details of the measures used for the present study are presented below.

### Sociodemographic and clinical characteristics

At baseline, patients provided information about their age, educational level (number of years of education), gender (male/female), living situation (with/without a spouse), having children (yes/no), and religiosity (yes/no/prefer not to specify). Ethnicity was measured by asking whether participants considered themselves a member of a minority ethnic group (yes/no). Healthcare providers gave information on patients’ type of cancer (lung/colorectal cancer), the time since diagnosis of the primary tumor, current stage of the disease, current treatment (yes/no receiving systemic treatment), and performance status according to the World Health Organization (WHO) (0-fully active to 3-capable of only limited self-care).

### Self-efficacy for participation in decision-making

To assess patient’s self-efficacy for participation in different activities associated with treatment-related decision-making, the decision-making participation self-efficacy scale (DEPS) was used [39]. Activities addressed in this scale include: (1) participating in discussions with the physician about available treatment options; (2) raising questions or concerns about the physician’s recommendations; (3) telling the physician which option one prefers; (4) resolving differences of opinion; (5) taking responsibility for making the final decision. Patients could score how confident they were about these items using a five-point Likert response scale, ranging from “not at all confident” to “completely confident”. The mean score of the responses to the five items was computed and then linearly transformed into a 0–100 metric scale score [39]. A higher score indicates a higher perceived self-efficacy for participation in treatment-related decision-making.

### Coping

Coping was measured using the subscales denial and acceptance of the COPE inventory and the subscales planning and active coping of the brief COPE [36]. Patients were asked to rate the items according to the best description of how they had been coping with their disease during the past 2 months. Items were rated on a four-point Likert scale from 1 (“I don’t do this at all”), 2 (“I do this a little bit”), 3 (“I do this a medium amount”), to 4 (“I do this a lot”). As recommended [40], principal components analysis was conducted to confirm subscales of the underlying coping strategies. The analysis confirmed the denial, acceptance, and problem-focused coping subscales. Responses per subscale were summed to create subscale sum scores. This resulted in a range of 4 to 16 for each subscale, with higher scores indicating more use of that particular coping strategy.

### Quality of life (QoL)

As overall quality of life was one of the variables of interest, we used a question from the EORTC QLQ-C30 questionnaire asking patients how they would rate their overall quality of life during the past week on a seven-point Likert scale ranging from 1 (poor) to 7 (excellent) [33].

### Patient satisfaction about care

As general patient satisfaction was one of the variables of interest, we used a question from the EORTC IN-PATSAT32 questionnaire, asking how patients, in general, would rate the hospital care they received during the past 2 months on a five-point Likert scale ranging from 1 (poor) to 5 (excellent) [38].

## Analyses

Descriptive statistics were used to summarize the characteristics of the study population. The Pearson *(r)* and Spearman (*r*_*s*_) correlation methods were used to, respectively, assess correlations of continuous and ordinal scale variables with self-efficacy scores. Multivariable linear regression analyses were performed to investigate the association of clinical characteristics, patient-reported outcomes (QoL and patient satisfaction with care), and coping strategies (independent variables) with patients’ self-efficacy (dependent variable). We used five linear regression models, with model 0 adjusted for sociodemographic characteristics including age, sex, living with a spouse, having children, years of education, religiosity, country of residence, and considering oneself a member of a minority group. We added the following variables to model 0: model (1a) clinical characteristics, model (1b) coping strategies, and model (1c) QoL and patient satisfaction with care. In the final model (model 2), all sociodemographic and clinical characteristics, patient-reported outcomes, and coping strategies were included. Regression coefficients and 95% confidence intervals were used to evaluate associations between the independent variables and self-efficacy. *P* values lower than 0.05 were considered statistically significant. The explained variance (adjusted *R*) of each model was shown.

Multiple imputation was used to deal with missing data of independent variables. Missing values on independent variables were imputed by the use of the multivariate imputation by chained equations (MICE) [41]. The percentages of missing values per variable varied from 0.2 to 14% and were assumed to be missing at random. Five imputed datasets were generated, and the results of the pooled analyses are presented. In sensitivity analysis, multivariable linear regression analyses were performed on cases with complete data, in order to assess departures from the missing at random assumption. All analyses were performed using SPSS Statistical version 28 (IBM).

## Results

The study population included 660 patients. The number of patients per country ranged from *n* = 25 in Slovenia to *n* = 164 in the Netherlands. Patients had a mean age of 66 years (SD 9.6), and the majority were men (*n* = 394, 60%). Most patients lived with a spouse (*n* = 490, 74%), and slightly more than half of the patients were religious (*n* = 334, 51%). Half of the patients were diagnosed with lung cancer (*n* = 331, 50%). Most patients received systemic anti-tumor treatment at the time of inclusion in the study (*n* = 581, 88%), (Table 1).

**Table 1:** Characteristics of the study population

	*n* = 660
Sociodemographic characteristics	
Age, *mean (SD)*	66.2 (9.6)
Missing*, n (%)*	3 (0.5)
Female sex, *n (%)*	266 (40.3)
Living with a spouse, *n (%)*	490 (74.2)
Missing*, n (%)*	17 (2.6)
Having children, *n (%)*	570 (86.4)
Missing*, n (%)*	7 (1.1)
Years of education, *mean (SD)*	12.9 (4.7)
Missing*, n (%)*	90 (13.6)
Religiosity, *n (%)*	
Religious	334 (50.6)
Not religious	223 (33.8)
Prefers not to specify	91 (13.8)
Missing*, n (%)*	12 (1.8)
Considering oneself member of minority group, *n (%)*	7 (1.1)
Missing*, n (%)*	25 (3.8)
Country of residence, *n (%)*	
Belgium	130 (19.7)
Denmark	68 (10.3)
Italy	136 (20.6)
Netherlands	164 (24.8)
Slovenia	25 (3.8)
UK	137 (20.8)
Clinical characteristics	
Diagnosis, *n (%)*	
Lung cancer	331 (50.2)
Colorectal cancer	329 (49.8)
Years since diagnosis, *mean (SD)*	1.7 (2.4)
Missing*, n (%)*	1 (0.2)
Years since diagnosis of current stage, *mean (SD)*	1.0 (1.4)
Missing*, n (%)*	2 (0.3)
Receiving systemic treatment, *n (%)*	581 (88.0)
Missing*, n (%)*	3 (0.5)
WHO performance status, *n (%)*	
3 in bed/ sitting for more than half of the day	8 (1.2)
2 up for more than half of the day	55 (8.3)
1 no heavy physical work	335 (50.8)
0 fully active	254 (38.5)
Missing*, n (%)*	8 (1.2)
Self-efficacy for participation in decision-making (DEPS, range 0-100), *mean (SD)*	72.8 (23.9)
Quality of Life (QoL) (EORTC QLQ-C15-PAL, range 1-7), *mean (SD)*	4.8 (1.3)
Missing*, n (%)*	7 (1.1)
Patient satisfaction (EORTC IN-PATSAT32, range 1-5), *mean (SD)*	4.0 (0.9)
Missing*, n (%)*	15 (2.3)

Patients had an average score of 72.8 (SD 23.9) on self-efficacy for participation in decision-making (Table 1, Supplemental Table 1). Patients in the Netherlands (B 10.77 (95% CI 5.47 to 16.07)), Denmark (B 10.34 (95% CI 3.53 to 17.15)), and UK (B 7.10 (95% CI 1.49 to 12.71)) had a higher level of self-efficacy for participation in decision-making compared to patients in Belgium (reference group), whereas Italian patients had a lower level ((B −7.38 (95% CI −12.94 to −1.83)) than patients in Belgium (model 0, not shown). Patients who indicated to be religious (B −4.33 (95% CI −8.41 to −0.25)) or preferred not to specify their religion (B −6.70 (95% CI −12.33 to −1.08)) had lower levels of self-efficacy for participation in decision-making compared to those who were not religious (model 0, not shown). In model 1a, a lower WHO performance status was associated with a lower level of self-efficacy for participation in decision-making (B −5.27 (95% CI −8.54 to −2.01)) (Table 2). Higher scores of problem-focused coping were associated with higher levels of self-efficacy for participation in decision-making ((B 1.63 (95% CI 0.96 to 2.31)) (model 1b). Higher scores for acceptance coping were also associated with higher levels of self-efficacy for participation in decision-making ((B 1.13 (95% CI 0.43 to 1.83)), (Table 2).

**Table 2:** The association between patient characteristics and self-efficacy in treatment-related decision-making (DEPS)

	Self-efficacy in decision-making			
	Model 1a^a^	Model 1b^b^	Model 1c^c^	Model 2^d^
	β (95% CI)	β (95% CI)	β (95% CI)	β (95% CI)
Sociodemographic and individual characteristics				
Age (years)	0.12 (−0.08–0.31)	0.16 (−0.03–0.35)	0.02 (−0.17–0.20)	0.10 (−0.08–0.29)
Sex				
Male	Reference	Reference	Reference	Reference
Female	2.01 (−1.76–5.77)	0.57 (−3.17–4.31)	2.18 (−1.39–5.75)	1.71 (−1.86–5.28)
Living with a spouse				
No	Reference	Reference	Reference	Reference
Yes	2.76 (−1.69–7.20)	3.36 (−0.97–7.68)	2.32 (−1.83–6.47)	1.37 (−2.81–5.54)
Having children				
No	Reference	Reference	Reference	Reference
Yes	−4.86 (10.32–0.59)	−3.25 (−8.73–2.23)	−4.38 (−9.54–0.78)	−3.43 (−8.67–1.82)
Years of education	0.18 (−0.21–0.58)	0.17 (−0.24–0.57)	0.34 (−0.05–0.73)	0.24 (−0.12–0.61)
Religiosity				
Not religious	Reference	Reference	Reference	Reference
Religious	**−4.34 (−8.38–−0.29)**	**−4.62 (−8.63– –0.60)**	−3.07 (−6.90–0.76)	−3.43 (−7.22–0.37)
Prefers not to specify	**−5.67 (−11.28 – –0.05)**	**−5.78 (−11.40– –0.16)**	−3.56 (−8.90 -1.77)	−2.79 (−8.09–2.51)
Considering oneself member of minority group				
No	Reference	Reference	Reference	Reference
Yes	−11.62 (−28.06–4.81)	11.55 (−29.95–6.85)	−7.86 (−23.91–8.20)	−7.10 (−27.38–8.50)
Country of residence				
Belgium	Reference	Reference	Reference	Reference
Denmark	**9.80 (2.96–16.65)**	4.38 (−2.49–11.24)	**11.62 (5.23–18.00)**	6.47 (−0.11–13.04)
Italy	**−8.19 (−13.74–2.64)**	**−11.94 (−17.67–−6.21)**	−2.29 (−7.58–3.00)	**−7.50 (−13.04–−1.96)**
Netherlands	**12.49 (7.01–17.96)**	5.10 (−0.45–10.65)	**12.54 (7.47–17.62)**	**7.85 (2.28–13.42)**
Slovenia	**13.16 (2.30–24.02)**	2.54 (−7.41–12.50)	**13.32 (3.90–22.75)**	9.49 (−0.85–19.84)
UK	**8.24 (2.58–13.89)**	1.76 (−4.00–7.53)	**8.00 (2.70–13.28)**	4.12 (−1.47–9.72)
Coping strategies				
Acceptance		**1.13 (0.43**–**1.83)**		0.67 (−0.01–1.33)
Problem focused		**1.63 (0.96–2.31)**		**1.41 (0.77**–**2.06)**
Denial		−0.10 (−0.67–0.48)		−0.15 ( −0.69–0.40)
Clinical characteristics				
Diagnosis				
Lung cancer	Reference			Reference
Colorectal cancer	−0.56 (−4.37–3.25)			0.61 (−2.99–4.20)
Years since diagnosis	0.63 (−0.13–1.39)			0.52 (−0.19–1.23)
Receiving systemic treatment				
No	Reference			Reference
Yes	1.20 (−4.73–7.13)			0.56 (−5.30–6.42)
WHO performance status (0 fully active–3 in bed/sitting for more than half of the day)	**−5.27 (−8.54−2.01)**			−2.15 (−5.24–0.94)
Patient died within 12 months after inclusion				
Yes	Reference			Reference
No	−0.50 (−5.35–4.35)			−2.33 (−7.28–2.62)
Patient reported outcome measures				
Quality of life (1 poor–7 excellent)			**2.78 (1.41**–**4.15)**	**2.34 (0.89–3.80)**
Patient satisfaction (1 poor–5 excellent)			**8.25 (6.29–10.22)**	**7.59 (5.61–9.56)**
Adjusted *R*^2^	0.12	0.17	0.23	0.28

Effect estimates marked with bold indicate statistically significant associations (*p* < 0.05)

^a^Model 1a: model 0 + clinical characteristics: diagnosis, years since diagnosis, receiving systemic treatment, WHO performance status, survival status for the first 12 months after inclusion

^b^Model 1b: model 0 + coping styles

^c^Model 1c: model 0 + patient-reported outcome measures

^d^Model 2: all variables

A better QoL was associated ((B 2.78 (95% CI 1.41 to 4.15)) with a higher level of self-efficacy for participation in decision-making. Greater patient satisfaction with hospital care was also associated ((B 8.25 (95% CI 6.29 to 10.22)) with a higher level of self-efficacy for participation in decision-making (model 1c). In the final model (model 2), the effect estimates for problem-focused coping style, QoL, patient satisfaction with care, and country of residence remained significant. The explained variance of this model was 28% (adjusted *R*^2^ = 0.28). The sensitivity analyses performed on a sample with complete data (*n* = 450) showed comparable results (Supplemental Table 2).

## Discussion

In this study in six European countries, we explored whether sociodemographic and clinical characteristics, coping strategies, and patient-reported outcomes were associated with self-efficacy for participation in treatment-related decision-making among patients with advanced cancer. We found problem-focused coping, better QoL, and more patient satisfaction with hospital care to be associated with higher levels of self-efficacy for participation in decision-making. Differences were observed in self-efficacy scores for participation in decision-making between countries.

The present study shows that patients reporting problem-focused coping strategies had a higher level of self-efficacy for participation in decision-making. In general, it is well known that patients reporting active or problem-focused coping fare better on a variety of physical and mental health outcomes than those less reporting active or problem-focused coping [42, 43]. Although literature on the association of coping and self-efficacy for participation in decision-making is scarce, our findings are in line with one study among slightly older patients showing that those who reported to have an active coping style rather than an avoidant coping style were more confident in interacting with physicians [14]. Patients who have an active or problem solving coping style may feel more confident to participate in decision-making with physicians, as they tend to show proactive behavior in order to solve issues as part of their coping strategy. A higher level of self-efficacy for participation in decision-making can lead to more participation in the decision-making process and, thus, positively influence health and functioning [15]. Therefore, supporting patients’ active coping mechanisms may lead to higher levels of self-efficacy for participation in decision-making about treatment.

In a study among patients with different types of cancer, those who reported to use active coping styles were more likely to have an active rather than passive role in decision-making [44]. In addition, it was suggested that patients’ decision-making role is one component of a coping style, indicating that decision-making role and coping style may be related as part of one process. This is in contrast to previous studies which considered decision-making and coping styles as separate phenomena [44]. It might be useful to study the role of self-efficacy for participation in decision-making in the context of different coping styles in order to gain a better understanding of self-efficacy for participation in decision-making.

It is worth noting that patients who prefer the doctor to make decisions do not necessarily have a lower level of self-efficacy for participation in decision-making. One study among 623 bladder, leukemia, and colorectal cancer survivors showed that those who prefer the physician to control decisions had similar self-efficacy levels for engaging in the decision-making process and greater trust in their physicians compared to those who prefer a more active role in decision-making [45]. This may illustrate the importance of supporting patients’ self-efficacy to participate in decision-making regardless of who controls the decisions. When physicians recognize and support patients’ capabilities and beliefs and establish a trusting relationship, physicians can help to foster self-efficacy beliefs regardless of patients’ preferences for an active or inactive role [46, 47]. Therefore, interventions to support or increase patients’ self-efficacy for participation during the decision-making process with the physician could facilitate the discussion about the extent to which patients wish to be engaged in the decision-making process [15]. This may particularly benefit patient populations characterized by low education or income, ethnic minorities, or low health literacy and, therefore, not optimally involved in treatment-related decision-making [4, 48].

Patient satisfaction with care was also associated with self-efficacy for participation in decision-making. Although comparable studies are lacking, one study among low-income men with early-stage prostate cancer also showed that less satisfaction with care was associated with lower self-efficacy to interact with physicians [20], and the same was found in a study of older patients [14]. Together with our results, these findings may imply that satisfaction with care can influence self-efficacy for participation in decision-making, or vice versa. Satisfaction with care often includes patients’ evaluation of the quality of communication with physicians [17]. A good patient-physician communication may lead to shared understanding, trust in decision-making, and, thus, more patient satisfaction [49, 50]. As a result, patients may feel valued, comfortable, and supported to ask questions and share concerns or expectations and, thus, develop a higher level of self-efficacy for participation in decision-making.

Better QoL was the third factor associated with a higher level of self-efficacy for participation in decision-making. It is unknown whether the association is due to patients’ physical or mental well-being. In a study among older persons, better health status was associated with a higher level of self-efficacy in patient-physician interactions [14]. In another study, patient self-efficacy in communicating with physicians predicted better QoL [20]. These studies imply that next to elements of the patient-physician interaction, self-efficacy for participation in decision-making can also affect and be affected by aspects of well-being. Given the impact of cancer on patients’ daily functioning and well-being, it is relevant that physicians and patients collaborate to discuss patients’ QoL and support patients to feel confident to engage in decision-making about their care and treatment.

Differences between countries were found in self-efficacy levels for participation in decision-making. Patients in the Netherlands had a higher level of self-efficacy for participation in decision-making than patients in Belgium, whereas Italian patients had a lower level than those in Belgium. In a previous European study, large variations were found between countries in the extent to which decisions were discussed with patients [51]. End-of-life decisions were discussed with the patient and relatives most frequently in the Netherlands and least frequently in Italy. For example, more than 50% of all end-of-life decisions were discussed neither with the patient nor with relatives in Italy. Patients were generally involved in decision-making in countries in which the frequency of making these decisions is high, which was the case in The Netherlands. Therefore, the practice of end-of-life discussions and patient involvement in countries may be associated with or reflect self-efficacy levels for participation in decision-making among patients and potentially explain differences between countries.

This study has several strengths. The DEPS scale used in this study covers several important aspects of self-efficacy for participation in decision-making such as the ability to ask questions. Other studies have used the validated perceived efficacy in patient-physicians interaction (PEPPI) scale [14] and the decision self-efficacy scale (DSE). However, these assess patients’ confidence in participating in interactions with physicians in general and not within the context of medical decision-making and do not provide emphasis on the decision-making process. Another strength is that we were able to include a substantial number of patients with advanced cancer across multiple countries, whereas data about patients at this stage are not easy to obtain due to their vulnerability.

One limitation of this study is that due to its cross-sectional design, it cannot provide robust insight into causal relationships. It is, however, likely that the associations studied are complex and bidirectional. In addition, it might be useful to understand which aspects of self-efficacy are particularly important in order to actually participate in decision-making [29]. Another limitation of this study relates to the low number of individuals considering themselves part of a minority group within the six countries. This might have led to sampling bias, i.e., people who participated to the study may reflect higher levels of self-efficacy for participation in decision-making compared to people from ethnic minority groups who may have lower levels of self-efficacy, for example, due to language barriers. Lastly, since we were interested in overall QoL and patient satisfaction in general, it remains unknown which elements of these concepts are related to self-efficacy for participation in decision-making. We only used one item measuring QoL and patient satisfaction. It would potentially have strengthened the reliability of results if we had used a multi-item scale measuring these constructs. Future research could investigate which specific components of quality of life and patient satisfaction correlate with self-efficacy for participation in decision-making. This would help to identify areas where interventions can be implemented more effectively.

Since our findings show associations of coping strategies, patient satisfaction, and quality of life with self-efficacy for participation in decision-making, we recommend to take into account these aspects when developing tailored interventions to facilitate self-efficacy or patient empowerment, especially in the face of a growing population of individuals living with advanced cancer. This requires physicians to become a skilled companion asking the right questions about what matters to the patient, while supporting them to actively participate in decision-making about treatment [52]. Given the potential benefits of a problem-focused coping style, it is important that healthcare professionals find patient-centered ways to support patients who have difficulties to cope with their disease [53]. Physicians should be aware that patients who have difficulties to cope with their disease or use avoidant coping strategies may not feel confident to interact with physicians. Also, adopting a participatory physician decision-making style in which patients are involved in the decision-making process may facilitate self-efficacy for participation in decision-making among patients [54].

In conclusion, this study shows that a problem-focused coping strategy, better patient satisfaction, and better QoL were associated with higher levels of self-efficacy for participation in decision-making among patients with advanced cancer. Differences between countries were observed in patients’ self-efficacy for participation in decision-making. We recommend physicians who aim to support patients in a process of decision-making to take into account patients’ coping strategy. Furthermore, they should be aware that patients’ self-efficacy for participation in decision-making is positively related to their satisfaction with care and their QoL.

## Supplementary information

ESM 1 (DOCX 22 kb)

## Data Availability

The dataset analyzed during the current study is not publicly available, but is available from the corresponding author on reasonable request.
