# Supplementary material for: Self-efficacy of advanced cancer patients for participation in treatment-related decision-making in six European countries: the ACTION study
Source: Support Care Cancer. 2023 Aug 8;31(9):512. doi: 10.1007/s00520-023-07974-2 (PMC10409662; doi:10.1007/s00520-023-07974-2)
Supplement: Supplementary file 1 — (DOCX 22 kb) [file 520_2023_7974_MOESM1_ESM.docx]

**SUPPLEMENTAL FILE**

**Supplemental Table 1.** Mean scores (SD) for self-efficacy for participation in decision-making (DEPS) and correlations of sociodemographic and clinical characteristics, patient reported outcomes and coping strategies with DEPS scores (N=660).

|  | **Self-efficacy for participation in decision-making (DEPS)** |
| --- | --- |
|  | Mean (SD),  Pearson’s r, Spearman’s r_s_ (scale variables) |
| **Sociodemographic and individual characteristics** |  |
| **Age (years)** | 0.03 |
| **Sex** |  |
| Male (n=394) | 72.4 (24.1) |
| Female (n=266) | 73.3 (23.7) |
| **Living with a spouse** |  |
| No (n=153) | 70.7 (23.6) |
| Yes (n=490) | 73.4 (24.0) |
| **Having children** |  |
| No (n=83) | 75.4 (22.2) |
| Yes (n=570) | 72.4 (24.2) |
| **Years of education** | 0.09 |
| **Religiosity** |  |
| Not religious (n=334) | 78.2 (22.6) |
| Religious (n=223) | 70.3 (24.6) |
| Prefers not to specify (n=91) | 68.3 (22.7) |
| **Considering oneself member of minority group** |  |
| No (n=628) | 72.9 (23.9) |
| Yes (n=7) | 59.4 (20.7) |
| **Country of residence** |  |
| Belgium (n=130) | 68.3 (28.3) |
| Denmark (n=68) | 80.4 (20.8) |
| Italy (n=136) | 60.4 (23.5) |
| Netherlands (n=164) | 80.2 (18.2) |
| Slovenia (n=25) | 75.8 (19.4) |
| United Kingdom (n=137) | 76.2 (22.5) |
| **Coping strategies** |  |
| Acceptance | 0.22 |
| Problem focused | 0.23 |
| Denial | -0.03 |
| **Clinical characteristics** |  |
| **Diagnosis** |  |
| Lung cancer (n=331) | 71.3 (23.5) |
| Colorectal cancer (n=329) | 74.2 (24.4) |
| **Years since diagnosis** | 0.07 |
| **Receiving systemic treatment** |  |
| No (n=76) | 73.8 (22.6) |
| Yes (n=581) | 72.6 (24.1) |
| **WHO performance status**  **(0 *fully active* – 3 *in bed/sitting for more than half of the day*)** | -0.08 (r_s_) |
| **Patient died within 12 months after inclusion** |  |
| Yes (n=226) | 72.6 (23.6) |
| No (n=340) | 72.9 (24.2) |
| **Patient reported outcome measures** |  |
| **Quality of life (1 *poor* – 7 *excellent*)** | 0.29 (r_s_) |
| **Patient satisfaction (1 *poor* – 5 *excellent*)** | 0.39 (r_s_) |

**Supplemental Table 2.** Complete case analysis on the association between patient characteristics and self-efficacy in treatment related decision-making (DEPS) (N=450).

|  | Self-efficacy in decision-making |  |  |  |
| --- | --- | --- | --- | --- |
|  | **Model 1a^a^** | **Model 1b^b^** | **Model 1c^c^** | **Model 2^d^** |
|  | β (95% CI) | β (95% CI) | β (95% CI) | β (95% CI) |
| **Sociodemographic and individual characteristics** |  |  |  |  |
| **Age (years)** | 0.03 (-0.20 – 0.26) | 0.05 (-0.17 – 0.27) | -0.00 (-0.21 - 0.20) | 0.09 (-0.12 - 0.30) |
| **Sex** |  |  |  |  |
| Male | Reference | Reference | Reference | Reference |
| Female | 0.63 (-3.75 – 5.01) | 0.70 (-3.61 – 5.00) | 2.11 (-1.92 - 6.14) | 1.84 (-2.16 – 5.84) |
| **Living with a spouse** |  |  |  |  |
| No | Reference | Reference | Reference | Reference |
| Yes | 0.82 (-4.45 – 6.09) | 2.78 (-2.40 – 7.95) | 1.97 (-2.84 - 6.77) | 1.92 (-2.93- 6.76) |
| **Having children** |  |  |  |  |
| No | Reference | Reference | Reference | Reference |
| Yes | -1.36 (-7.54 – 4.83) | -0.92 (-7.22 - 5.38) | -2.84 (-8.70 – 3.02) | -2.72 (-8.48 – 3.05) |
| **Years of education** | 0.23 (-0.25 – 0.70) | 0.15 (-0.32 – 0.62) | **0.44 (0.01 – 0.87)** | 0.20 ( -0.22 – 0.64) |
| **Religiosity** |  |  |  |  |
| Not religious | reference | Reference | Reference | Reference |
| Religious | -2.63 (-7.17 – 1.91) | -3.77 (-8.25 - 0.71) | -1.71 (-5.90 – 2.47) | -2.09 (-6.23 – 2.05) |
| Prefers not to specify | -3.19 (-9.84 – 3.46) | -2.80 (-9.25 – 3.64) | -2.33 (-8.36 – 3.69) | -1.23 (-7.20 – 4.73) |
| **Considering oneself member of minority group** |  |  |  |  |
| No | Reference | Reference | Reference | Reference |
| Yes | -7.00 (-28.86 – 14.86) | -5.33 (-26.47 – 15.81) | -8.62 (-28.27 – 11.03) | -5.34 (-24.69 – 14.01) |
| **Country of residence** |  |  |  |  |
| Belgium | Reference | Reference | Reference | Reference |
| Denmark | **9.13 (1.66 -16.60)** | 2.88 (-4.60 – 10.36) | **10.01 (3.20 – 16.81)** | 6.27 (-0.76 - 13.30) |
| Italy | **-10.34 (-17.48 – -3.20)** | **-14.67 (-22.00 – -7.35)** | -4.34 (-11.04 – 2.36) | **-8.29 (-15.24 - -1.34)** |
| Netherlands | **10.33 (4.01 -16.64)** | 2.69 (-3.73 – 9.10) | **10.53 (4.70 – 16.36)** | **7.54 (1.29 – 13.80)** |
| Slovenia | 12.55 (-1.33 – 26.44) | 0.10 (-12.72 – 12.92) | **13.25 (1.38 – 25.12)** | 9.71 (-3.03 – 22.45) |
| United Kingdom | 6.84 (-0.12 – 13.80) | 1.31 (-5.63 – 8.25) | **7.39 (1.00 – 13.78)** | 4.67 (-2.02 – 11.35) |
| **Coping strategies** |  |  |  |  |
| Acceptance |  | **1.11 (0.29 - 1.92)** |  | 0.68 (-0.07 - 1.43) |
| Problem focused |  | **1.80 (-1.02 – 2.57)** |  | **1.49 (0.77- 2.21)** |
| Denial |  | 0.08 (-0.62 - 0.78) |  | 0.02 (-0.63 – 0.66) |
| **Clinical characteristics** |  |  |  |  |
| **Diagnosis** |  |  |  |  |
| Lung cancer | Reference |  |  | Reference |
| Colorectal cancer | -0.13 (-4.61 – 4.36) |  |  | 1.58 (-2.53 – 5.70) |
| **Years since diagnosis** | 0.09 (-0.99 - 1.17) |  |  | 0.25 (-0.73 – 1.22) |
| **Receiving systemic treatment** |  |  |  |  |
| No | Reference |  |  | Reference |
| Yes | -0.42 (-8.09 – 7.24) |  |  | 1.54 (-5.59 – 8.66) |
| **WHO performance status**  **(0 *fully active* – 3 *in bed/sitting for more than half of the day*)** | **-6.67 (-10.36 – -2.99)** |  |  | -2.70 (-6.15 - 0.76) |
| **Patient died within 12 months after inclusion** |  |  |  |  |
| Yes | Reference |  |  | Reference |
| No | -1.24 (-5.68 – 3.20) |  |  | -3.72 (-7.81 - 0.37) |
| **Patient reported outcome measures** |  |  |  |  |
| **Quality of life (1 *poor* – 7 *excellent*)** |  |  | **4.15 (2.50 - 5.80)** | **3.67 (1.94 – 5.39)** |
| **Patient satisfaction (1 *poor* – 5 *excellent*)** |  |  | **8.59 (6.26 - 10.93)** | **7.75 (5.45 – 10.06)** |
| ***Adjusted R^2^*** | 0.10 | 0.14 | 0.25 | 0.29 |

Effect estimates marked with bold indicate statistically significant associations (p<0.05).

^a^ Model 1a: Model 0 + clinical characteristics: diagnosis, years since diagnosis, receiving systemic treatment, WHO performance status, survival status for the first 12 months after inclusion.

^b^ Model 1b: Model 0 + coping styles.

^c^ Model 1c: model 0 + patient reported outcome measures.

^d^ Model 2: all variables.
